# Characterization of Self-Powered Triboelectric Tachometer with Low Friction Force

**DOI:** 10.3390/mi12121457

**Published:** 2021-11-27

**Authors:** Ling Bu, Xinbao Hou, Lanxing Qin, Zhiwei Wang, Feng Zhang, Feng Li, Tao Liu

**Affiliations:** School of Information Engineering, China University of Geosciences, Beijing 100083, China; 2004190005@cugb.edu.cn (X.H.); qlx0503@163.com (L.Q.); 1004182214@cugb.edu.cn (Z.W.); zfeng7975@cugb.edu.cn (F.Z.); 2104200005@cugb.edu.cn (F.L.); 1004182222@cugb.edu.cn (T.L.)

**Keywords:** self-powered, triboelectric, tachometer, low friction force

## Abstract

Self-powered triboelectric tachometers have wide application prospects in mechanical and electrical industries. However, traditional disc-type tachometers typically require large contact force, which burdens rotary load and increases frictional wear. To reduce the friction force of triboelectric tachometers, we present an alternative structure defined by flapping between rigid and flexible triboelectric layers. In this work, we further characterize this type of tachometer, with particular focus on the oscillating relationship between output voltage and rotation speed due to the plucking mechanism. This oscillating relationship has been demonstrated both theoretically and experimentally. For future self-powered triboelectric tachometers, the proved oscillating relationship can be applied as calibration criteria for further enhancing sensitivity and linearity in rotation measurement.

## 1. Introduction

In the Internet of Things era, massive sensing equipment is indispensable for the realization of intelligent buildings, transportation, cities, etc. [1]. Accompanying the large quantity of sensors in versatile environments is the equally massive demand of distributed and long-lasting power sources, which can hardly be satisfied by traditional powering methods like batteries or cables. Toward this demand, triboelectric nanogenerators present a promising power solution, as they can convert ambient tribology-related energy to electricity with quite high voltage, power, and efficiency [2,3,4,5]. Moreover, triboelectric nanogenerators can be inherently incorporated into sensors to construct self-powered devices [6,7,8], which further alleviates the powering issue and has already found numerous applications in wearable electronics [9,10,11], blue energy [12,13,14], infrastructure monitoring [15,16,17], etc.

Tachometers are widely needed in mechanical and electrical industries to characterize or monitor the rotation speed of shafts, gears, motors, etc. [18]. Constructing a self-powered tachometer is of huge industrial application interest. So far, there have been plenty of disc-based tachometers reported [19,20,21,22,23], which typically consist of a rotating disc with constant rotation speed and a fixed disc facing the rotating one. Because of the tight contact and large contact area, the friction force between the two discs is very high, and the triboelectric output is usually of high sensitivity. However, the large friction force also adds up to the load of the rotating disc, causing potential systematic errors in rotation speed characterization and increasing frictional wear of the measuring system in long-term monitoring [24,25]. Further, for most industrial applications, the revolving parts prefer measurement with less or even no contact, which renders it difficult to directly apply a disc-type tachometer to certain established industrial scenarios.

To reduce the frictional force of the triboelectric tachometer, Wang et al. designed a rotating sensor with polymer films connected to the shaft, sweeping and sliding through adjacent electrodes [26]. This design achieves ultra-low friction force, but the output performance is limited compared with the simultaneously incorporated electromagnetic part. Zhang et al. proposed a non-contact cylindrical rotating triboelectric nanogenerator, but it required charge injection pre-treatment of the materials to guarantee output, and is actually based on the electrostatic induction principle [27]. Wu et al. applied the low frictional force triboelectric tachometer to the turbodrills with a structure similar to [26], but tested nonlinear voltage versus rotation speed relations [28]. Lin et al. rationally designed the rotation triboelectric nanogenerators to extend lifetime and durability by combining the rotating shaft with a spring, so that the contact force between the two rotating discs can be adjusted to a proper value through spring restoring force [29]. Previously, we have presented an alternative structure of a rotary triboelectric nanogenerator based on flapping between rigid and flexible triboelectric layers [30], which is based on the plucking mechanism, as a by-standing flexible blade is periodically perturbed by a rigid blade secured to the rotating parts. Because of the large deformation capability of the flexible blade, both contact force and friction force are significantly reduced, and tachometers based on this structure can be more readily applied in industrial scenarios. However, the output of this type of tachometers still suffers from the problem of accuracy and linearity, as the plucking mechanism dictates that the flexible blade experiences oscillations after one contact, and can be deviated from the equilibrium position. In this context, the voltage versus rotation speed relationship needs to be amended to reduce the possible influences of voltage fluctuation and to improve the tachometer’s accuracy and linearity. Therefore, in this work we present detailed characterization of the voltage versus rotation speed relationship both theoretically and experimentally, which is the foremost step toward the precise and practical calibration criteria for self-powered triboelectric tachometers.

## 2. Model

**Figure 1:**
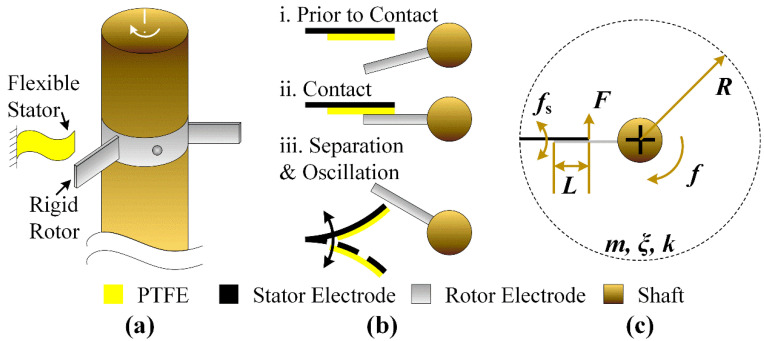
Triboelectric tachometer with low friction force: (**a**) Configuration; (**b**) Dynamic mechanism; (**c**) Model plot.

The configuration of the low friction force triboelectric tachometer is shown in Figure 1a. To measure the rotation speed of the revolving shaft, a rigid rotor electrode is fixed onto the shaft, and a flexible stator is clamped at one end beside the shaft. This compact design eliminates the requirement of face-to-face adding, compressing and sliding the triboelectric layer with respect to the revolving surface, and is suitable for most application scenarios, especially determining the rotation speed of bearings and gears in machine tools. Moreover, the contact mechanism between rigid and flexible parts considerably reduces frictional force and wear, owing to the large deformation capability of the flexible stator. In practice, the flexible stator is clamped at a proper height to guarantee full contact area with respect to the rotor. Figure 1b shows the dynamics of the contact mechanism between the rigid and flexible parts. Suppose that the flexible stator is in the equilibrium state (both deflection and velocity equal zero), and the rigid rotor is gradually approaching from the (i) Prior to Contact state. Upon (ii) Contact state, charge pairs are generated at the interface between the polytetrafluoroethylene (PTFE) layer on the stator electrode and the rotor electrode, producing a voltage spike on external load. Thereafter is the (iii) Separation & Oscillation state, where the rigid rotor passes through and the flexible stator starts self-oscillation at its natural frequency. It is this self-oscillation process that results in the output fluctuation of the triboelectric tachometer. As the flexible stator exhibits varying deflection and velocity in the self-oscillation process, the original equilibrium state cannot be guaranteed for the next contact, and this results in change of actual deflection state and, thus, output voltage. It can be seen that the varying output voltage is deeply correlated with the self-oscillation characteristics of the flexible stator; therefore, in subsequent work we mainly investigate how the relation between natural frequency of the stator (*f*_s_) and the revolving frequency of the rotor (*f*) affects triboelectric tachometer output. These two parameters are also presented in terms of self-oscillation cycle (*T*_s_) and revolving cycle (*T*), as *T*_s_ = 1/*f*_s_ and *T* = 1/*f*.

Figure 1c plots the simplified model of the triboelectric tachometer. Apart from the two key parameters *f*_s_ and *f* denoted above, we also adopted parameters including: mass of flexible stator (*m*); spring contact of flexible stator (*k*); and damping ratio (*ξ*). For ease of calculation, contact force (*F*) between rigid rotor and flexible stator is assumed to be of constant value and constant duration of *t*_c_. *R* and *L* are the geometry parameters, in which *R* is the radius from the revolving shaft to the flexible stator, and *L* is the overlapped length between rigid and flexible parts. Both *R* and *L* are assumed to be constant to guarantee constant force (*F*).

The dynamics of the flexible stator is governed by Equation (1):

(1) $$m\ddot{x}+\xi \dot{x}+kx=\{\begin{array}{l} FnT\leq t\leq nT+t_{c}\\ 0nT+t_{c}\leq t\leq \left(n+1\right)T \end{array}$$

where *x* is the displacement of the tip of the flexible stator, and *n* is arbitrary integer.

Equation (1) implicitly incorporates the key parameter *f* in terms of *T*, and the key parameter *f*_s_ in that:

(2) $$f_{s}=\frac{1}{2\pi }\sqrt{\frac{k}{m}}$$

**Figure 2:**
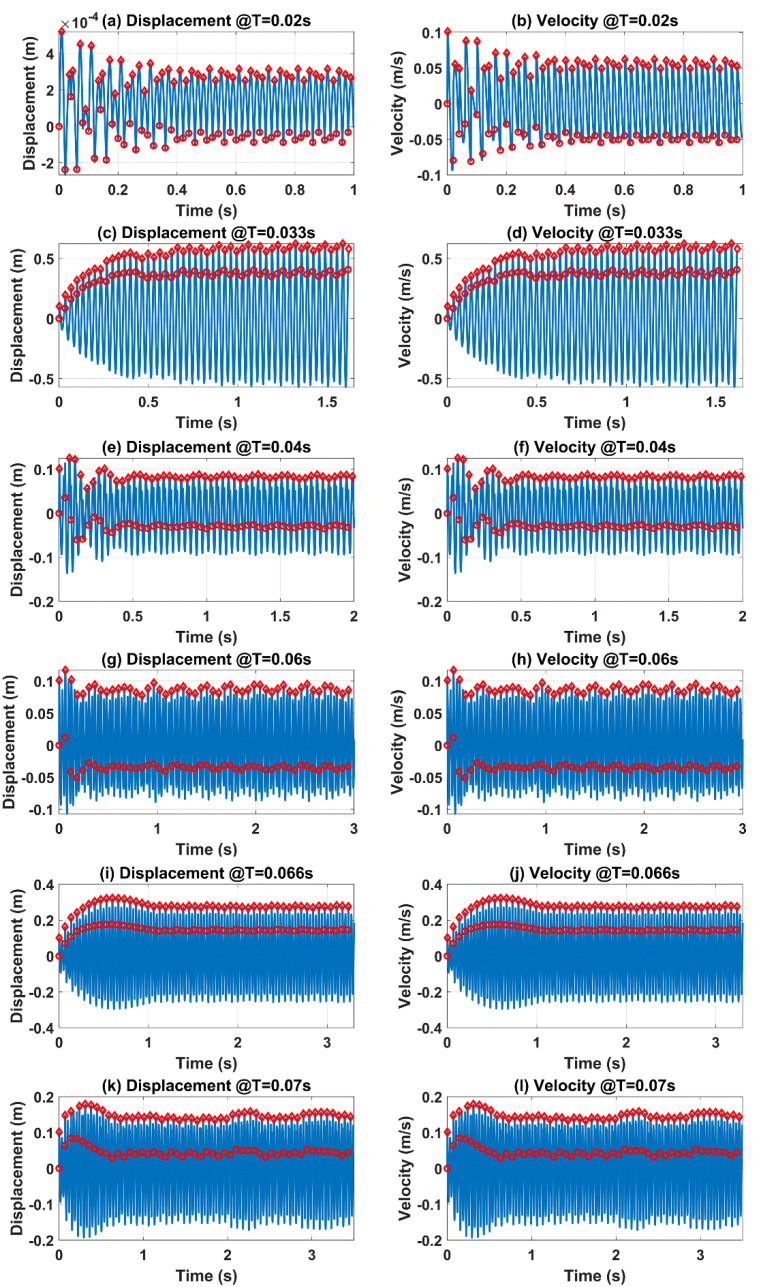
Calculated time traces of displacement (**a**,**c**,**e**,**g**,**i**,**k**) and velocity (**b**,**d**,**f**,**h**,**j**,**l**) at different rotation speeds.

Based on the above two equations, the actual deflection state of the flexible stator can be described by tip displacement and velocity. Through updating the actual deflection state as the original state of Equation (1) before each contact, the dynamic characteristics of the flexible stator at different *T* can be derived. Based on experimentally tried and true values, we adopted specific parameters as: *m* = 0.01 kg; *k* = 350 N/m; *ξ* = 0.1; *F* = 1 N; and *t*_c_ = 1 ms. In this way, we set *f*_s_ to be a constant value of 29.79 Hz, i.e., the self-oscillation cycle of the flexible stator was *T*_s_ = 0.033 s, but we swept the revolving cycle *T* to obtain varying relations between *f*_s_ and *f*. Figure 2 shows the calculated results of the flexible stator tip displacement and velocity at different *T*. The general trend shows that, when *T* ≤ *T*_s_, only one cycle of tip displacement or velocity change was obtained for each contact; but when *T* > *T*_s_, there was more than one cycle of tip displacement or velocity change for each contact, indicating that self-oscillation was activated. For the six different *T* values, 100 iterations of contacts were calculated. In Figure 2, only the first 50 contacts were plotted for ease of observation. It can be seen that the transient processes completed within the first 50 contacts, and the tip displacement and velocity could maintain stability after 50 contacts. For each contact, the initial condition is marked using dots, and the maximal result is marked using diamonds. The varying dot and diamond positions in all 12 subplots demonstrate that both the initial conditions and the maximal results changed for each contact.

To quantify the influence of the initial condition on tip displacement and velocity for each contact, we denoted the following two parameters:

(3) $$\Delta d_{n}=d_{\max,n}-d_{0,n}$$

(4) $$\Delta v_{n}=v_{\max,n}-v_{0,n}$$

where *d*_max,n_ is the diamond maximal tip displacement after the *n*th contact, and *d*_0,n_ is the dotted initial tip displacement for the *n*th contact, so that Δ*d*_n_ reflects maximal displacement change caused by the initial condition of the *n*th contact. Similarly in Equation (4), *v*_max,n_ and *v*_0,n_ are the diamond maximal tip velocity and dotted initial tip velocity for the *n*th contact, and Δ*v*_n_ reflects maximal velocity change caused by the initial condition of the *n*th contact.

With the help of Δ*d*_n_ and Δ*v*_n_, more generalizations could be obtained from Figure 2 in terms of the maximal change of tip displacement and velocity in each contact. Taking maximal change of tip displacement as an example: in Figure 2a, when *T* = 0.02 s, Δ*d*_n_ first went through a transient process, and then gradually decreased to reach a stable value. Similar situations applied for *T* = 0.04 s and *T* = 0.07 s, as can be seen from Figure 2e,k. On the contrary, in Figure 2c, when *T* = 0.033 s, Δ*d*_n_ also experienced a settling process, but in an increasing trend, before finally becoming stable. Similar observations can be made for *T* = 0.06 s and *T* = 0.066 s, as is shown in Figure 2g,i. Maximal change of tip velocity can also be observed in similar way. Although the two generalizations can be clearly observed, it is still necessary to further quantify the decrease or increase extent of Δ*d_n_* and Δ*v_n_* for comparison, so as to obtain a clear trend of Δ*d*_n_ and Δ*v*_n_ versus varying *T* values.

To further compare Δ*d*_n_ and Δ*v*_n_ for different *T* values, normalization process is carried out as:

(5) $$\Delta d_{n}^{NORM}=\frac{\Delta d_{n}}{\Delta d_{1}}$$

(6) $$\Delta v_{n}^{NORM}=\frac{\Delta v_{n}}{\Delta v_{1}}$$

where Δ*d*_1_ and Δ*v*_1_ are the maximal displacement and velocity change caused by the initial condition of the 1st contact, i.e., caused by the equilibrium state. The normalized parameters $\Delta d_{n}^{NORM}$ and $\Delta v_{n}^{NORM}$ excludes the incomparable magnitude of displacement and velocity change at different *T* values in the form of ratios, so that they can be compared as a unit-less number versus 1, and can be characterized if the displacement and velocity change subject to current initial values outperforms or underperforms that subject to equilibrium state for one contact.

**Figure 3:**
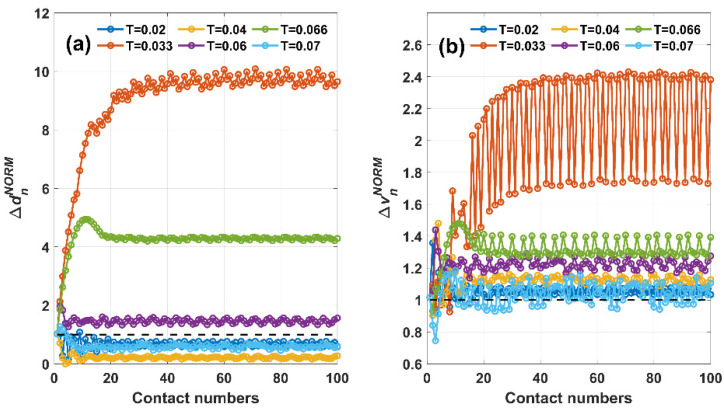
Normalized ratio vs. contact numbers: (**a**) displacement; (**b**) velocity.

Figure 3 shows the quantified comparison of Δ*d*_n_*^NORM^* and Δ*v*_n_*^NORM^* for the above six different *T* values in all 100 contacts. In Figure 3a, all six curves of Δ*d*_n_*^NORM^* start from the same point of Δ*d*_1_*^NORM^* = 1, but two clear distinctions are exhibited after the settling process. For *T* = 0.033 s, 0.06 s, and 0.066 s, all three curves of Δ*d*_n_*^NORM^* exceeded the black dashed line of 1, while for *T* = 0.02 s, 0.04 s, and 0.07 s, all three curves of Δ*d*_n_*^NORM^* fell below the same dashed line of 1. This is in accordance with previous two generalizations observed in Figure 2. In addition, the normalized parameter Δ*d*_n_*^NORM^* enables further comparison for different *T* values, as after stabilization *T* = 0.033 s generated maximal Δ*d*_n_*^NORM^* and *T* = 0.04 s generated the minimal Δ*d*_n_*^NORM^*. For *T* = 0.02 s and *T* = 0.07 s, the stable Δ*d*_n_*^NORM^* values were very much close. Figure 3b shows the six curves of Δ*v*_n_*^NORM^*, which are, however, quite different when compared with Figure 3a. Though all six curves still started from the same point of Δ*v*_1_*^NORM^* = 1, all six curves exceeded the black dashed line of 1. *T* = 0.033 s still generated maximal Δ*v*_n_*^NORM^*, but both *T* = 0.02 s and *T* = 0.07 s generated the minimal Δ*v*_n_*^NORM^*. This means that the distinction in terms of Δ*v*_n_*^NORM^* was not as clear as that of Δ*d*_n_*^NORM^*. Moreover, for almost all six curves of Δ*v*_n_*^NORM^*, the deviation between contacts was much more severe compared with that of Δ*d*_n_*^NORM^*, indicating that Δ*v*_n_*^NORM^* is less as favorable than Δ*d*_n_*^NORM^* in the dynamic characterization of triboelectric tachometers.

**Figure 4:**
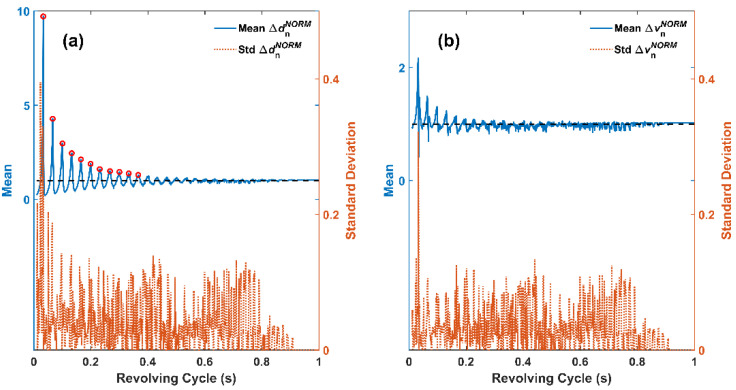
Mean and standard deviation of (**a**) displacement and (**b**) velocity versus revolving cycle.

In order to compare the steady state values of Δ*d*_n_*^NORM^* and Δ*v*_n_*^NORM^*, Figure 4 summarizes the mean and standard deviation of Δ*d*_n_*^NORM^* and Δ*v*_n_*^NORM^* versus varying *T* values. For each *T* value, both mean and standard deviation of Δ*d*_n_*^NORM^* and Δ*v*_n_*^NORM^* are calculated for the 51–100 contacts, so as to eliminate the effect of settling process. In Figure 4a, the blue curve represents mean Δ*d*_n_*^NORM^*, and the red curve represents standard deviation of Δ*d*_n_*^NORM^*. Interestingly, it can be seen that when *T* was small (i.e., comparable to stator cycle *T*_s_), mean Δ*d*_n_*^NORM^* followed an oscillating style as *T* gradually increased, and the peaks of this oscillation decreased in this process, as is summarized in Table 1. When *T* was sufficiently large (*T* > 0.66 s, i.e., cycle ratio *T*/*T*_s_ > 20), such oscillation disappeared, and the mean Δ*d*_n_*^NORM^* maintained a stable value of 1, as is plotted using the black dashed line in Figure 4a. This can be explained as follows: (1) When *T* is small, the influence of *T*_s_ cannot be neglected, as the flexible stator might self-oscillate to a particular deflection state other than the equilibrium state when contacts occur, and the particular deflection state changes the attainable tip displacement; (2) when *T* is sufficiently large, the flexible stator has sufficiently long time to recover from last contact. So, when next contact occurs, the flexible stator has resumed equilibrium state, and therefore Δ*d*_n_ in each contact equals Δ*d*_1_, i.e., Δ*d*_n_*^NORM^* equals 1. This implies that a triboelectric tachometer can obtain accurate results for low rotation speed, but need additional calibration for high rotation speed. Additionally, the 11 highest decreasing peaks in the mean Δ*d*_n_*^NORM^* curve have been dotted in Figure 4a and listed in Table 1. It can be seen that all these peaks occur when *T* is integer times of *T*_s_, and all these peak values exceed 1. In terms of standard deviation, when *T* < 0.9 s (i.e., cycle ratio *T*/*T*_s_ < ~30), the standard deviation of Δ*d*_n_*^NORM^* cannot be neglected, indicating the existence of steady state fluctuation. The maximal standard deviation of 0.4 occured when mean Δ*d*_n_*^NORM^* reached maximum, and for most other occasions the standard deviation was less than 0.18. When *T* > 0.9 s, the standard deviation was zero, indicating that no steady state fluctuation existed and the triboelectric tachometer generated very stable output. Figure 4b plots the mean and standard deviation curves of Δ*v*_n_*^NORM^* versus varying *T* values, and similar trend can be observed as compared to Figure 4a. When *T* was small, the mean Δ*v*_n_*^NORM^* also exhibited oscillation with decreasing peaks as *T* gradually increased. When *T* was sufficiently large, the mean Δ*v*_n_*^NORM^* also arrived at the stable value of 1 (black dashed line). However, the mean Δ*v*_n_*^NORM^* curve contained more burrs than the mean Δ*d*_n_*^NORM^* curve, indicating that finding the calibration criteria from the perspective of Δ*v*_n_*^NORM^* would be more difficult and less ideal than from that of Δ*d*_n_*^NORM^*. Still, the maximal standard deviation of 0.32 occured when mean Δ*v*_n_*^NORM^* reached maximum, and for most other occasions the standard deviation was approximately 0.1. Again, when *T* > 0.9 s, the standard deviation was zero, indicating that the flexible stator was of stable velocity change. The interesting oscillation characteristics of the flexible stator shown in Figure 4 is worth further experimental validation, and following experiments mainly focus on this point.

## 3. Experiments

**Figure 5:**
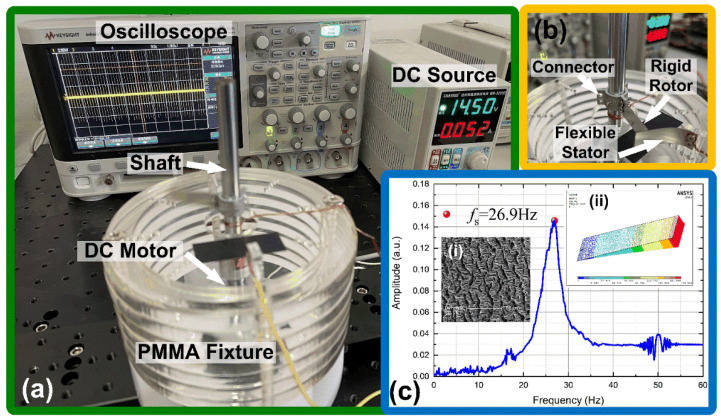
Experimental setup. (**a**) Photo of testing rigs; (**b**) Close-up photo of flexible stator and rigid rotor; (**c**) Frequency sweep test result of the flexible stator, showing resonant frequency of 26.9 Hz. Inset (i) in (**c**) is the scanning electron microscopy (SEM) photo of surface morphology of polytetrafluoroethylene (PTFE) film on the flexible stator, and Inset (ii) is the modal finite element analysis result of the flexible stator, showing theoretical resonant frequency of 28.33 Hz.

Figure 5 shows the experimental setup to verify the triboelectric tachometer. For the testing rigs shown in Figure 5a, the DC motor and the circular fixtures were secured on the testing platform. The rigid rotor was fixed to the motor shaft and electrically grounded. The rotation speed of the motor was controlled by a DC voltage source (Zhaoxin MN-3205D, Shenzheng, China). The flexible stator was fastened beside the motor shaft on the circular fixtures, and the sensing signal on the flexible stator electrode was directly measured using the oscilloscope (Keysight InfiniiVision DSOX2024A, Santa Rosa, CA, USA) with 1 MΩ input resistance. Figure 5a is a snapshot in the revolution process of the motor shaft, which caused blurring of the rigid rotor and flexible stator. To show this clearly, in Figure 5b we present the close-up photo, in which the rigid rotor is connected to the motor shaft via a specialized connector, and the flexible stator bends in the contact process. The dimension of both the rigid rotor and the flexible stator was 2.95 cm × 10 cm, and the overlapped length between the rotor and the stator was 1.8 cm. Both the rotor and the stator were made of stainless steel with thicknesses of 1 mm and 0.05 mm, respectively. PTFE thin film with 90 μm thickness (Spider (Amoy) S&T Co., Ltd., Xiamen, China) was stuck to the flexible stator, so altogether the flexible stator was 0.14 mm in thickness. For wiring, the rotor was grounded, and Figure 5c shows the measured frequency response of the flexible stator using the locked-in amplifier (Stanford Research Systems SR865, Sunnyvale, CA, USA), and the measured resonant frequency was 26.9 Hz. Inset (i) of Figure 5c is the micro-slits featured surface morphology of the PTFE film to enhance triboelectricity, and the scale bar length is 50 μm. Inset (ii) is the finite element modal analysis of the flexible stator using ANSYS Version 14.5, and the simulated first mode resonant frequency was 28.33 Hz for the proposed geometry, which was very close to the measured resonant frequency and the *f_s_* value adopted in previous calculation.

**Figure 6:**
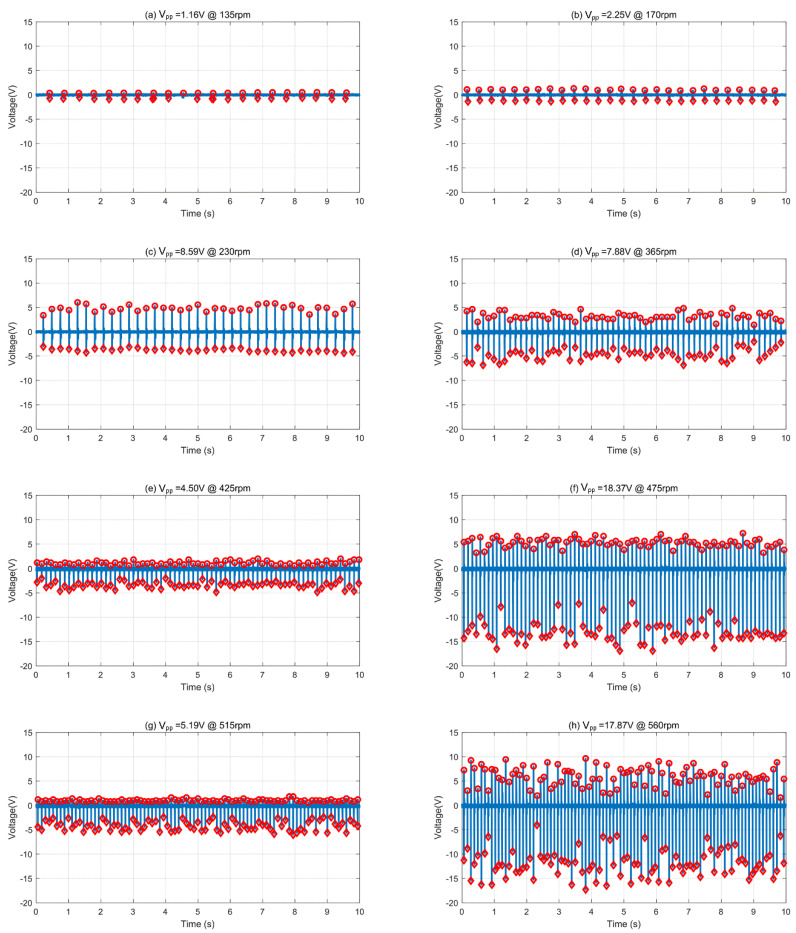
Measured voltage–time curves at different rotation speeds.

By varying the rotation speed of the motor shaft, the sensing signals on the stator electrode were measured and shown in Figure 6. Altogether we measured 43 different rotation speeds spanning from 45 revolutions per minute (rpm) to 560 rpm, as these two values were the experimentally attainable lowest and highest rotation speeds of our DC motor. In Figure 6, 8 of the 43 different rotation speeds are picked out as an illustration, namely 135 rpm, 170 rpm, 230 rpm, 365 rpm, 425 rpm, 475 rpm, 515 rpm, and 560 rpm. The corresponding revolving frequency for these 8 rotation speeds was 2.25 Hz, 2.83 Hz, 3.83 Hz, 6.08 Hz, 7.08 Hz, 7.92 Hz, 8.58 Hz, and 9.33 Hz, and the respective revolving cycle decreased from 0.44 s, 0.35 s, 0.26 s, 0.16 s, 0.14 s, 0.13 s, 0.12 s, to 0.11 s. At all rotation speeds, the triboelectric voltage-time curves were recorded, and the general trend was a series of voltage spikes corresponding to a series of contacts. In this work, the peak-to-peak voltage (*V*_pp_) of these voltage spikes was adopted to characterize the triboelectric tachometer output, so as to avoid the possible positive and negative voltage asymmetry and to better reflect the total triboelectric output voltage generated at different rotation speeds. In all subplots of Figure 6, the maximal positive triboelectric voltage for all contacts is marked using dots, and the minimal negative triboelectric voltage is marked using diamonds. *V*_pp_ for each contact was calculated as the difference between the maximal and minimal voltage value, and the measured multiple *V*_pp_ values were averaged over the same testing period of 10 s for each rotation speed. Throughout the subplots, it can be seen that the averaged *V*_pp_ values were 1.16 V, 2.25 V, 8.59 V, 7.88 V, 4.50 V, 18.37 V, 5.19 V, and 17.87 V for the above rotation speeds, demonstrating that no simple linear relationship existed for generated voltage and rotation speed. Rather, the generated voltage also exhibits in an oscillating fashion as the rotation speed increases.

**Figure 7:**
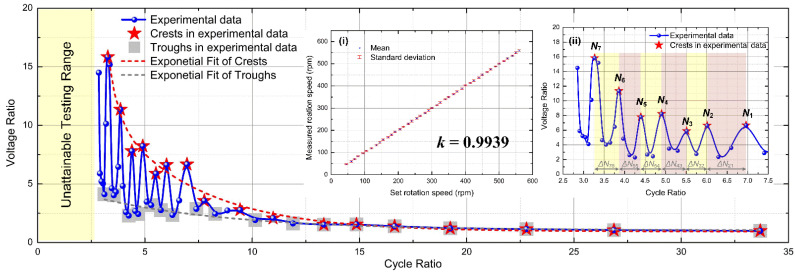
Characterization of measured voltage ratio vs. cycle ratio, with inset (**i**) sensitivity of the triboelectric tachometer based on time measurement of voltage spikes; (**ii**) close-up of the voltage ratio oscillation in the range of 2.5–7.5 cycle ratio.

To better clarify the relation between the triboelectric voltage of the tachometer and rotation speed, Figure 7 further plots the relation of voltage ratio versus cycle ratio based on five sets of voltage data measured for all 43 rotation speeds. In Figure 7, the vertical axis of voltage ratio is defined as the ratio of voltage at specific rotation speed over voltage at 45 rpm. In other words, because 45 rpm represents a relatively long revolving cycle for the flexible stator to resume its equilibrium state, this voltage ratio can also be understood as the normalized voltage with respect to the equilibrium state. The horizontal axis of cycle ratio is defined as the ratio of rotor revolving cycle *T* over stator self-oscillation cycle *T*_s_, or the reciprocal of rotor revolving frequency 1/*f* multiplied by stator resonant frequency *f*_s_. In other words, this cycle ratio equals *T*/*T*_s_ or *f*_s_/*f*. For all 43 datapoints averaged over five sets, the voltage ratio showed an oscillating style with respect to the cycle ratio. In Figure 7, all crests in the 43 datapoints are marked using pentagons, and all troughs are marked using squares. Both the crests and the troughs can be fitted exponentially, and these two exponentially fitted lines constitute the upper and lower limit of the experimental datapoints. For the tachometer function, the oscillating part of the curve in Figure 7 (i.e., cycle ratio is less than 15) could serve as the calibration criteria for measuring and characterizing rotation speed of the motor shaft. When the revolving cycle to be measured was sufficiently large, i.e., cycle ratio is greater than 15, the oscillation feature disappeared as both crests and troughs coincided with the datapoints. In this condition, the voltage ratio and cycle ratio curve could be degraded into a unified exponential curve. Apart from the voltage characterization method, the inset (i) in Figure 7 shows an alternative method of measuring the rotation speed via averaging the timespan between triboelectric voltage spikes, which also shows good sensitivity and linearity. The slope of curve between measured rotation speed and set rotation speed was 0.9939, which is very close to 1.

Inset (ii) in Figure 7 shows the close-up of the highest seven crests, which are marked as *N*_1_ to *N*_7_ from right to left. This close-up shows the oscillating style with decreasing peaks more clearly. However, further characterization of the distance between these seven crests indicated a slight deviation from the theoretical model. The distance between adjacent crests Δ*N_ij_* is defined as:

(7) $$\Delta N_{ij}=\Delta N_{j}-\Delta N_{i}i>j\in [1,2,\ldots ,7]$$

and the results of the six Δ*N_ij_* are shown in Table 2. It can be seen that the distances between the peaks actually decreased as cycle ratio reduced, and the peaks did not necessarily occur at integer times of the stator self-oscillation cycle *T*_s_ as shown in Table 1. This deviation is possibly due to the discrepancy with previous model assumption, as the duration of contact force *F* between rigid rotor and flexible stator may also have fluctuated. Future works can concentrate on this point to present a more detailed specification of the oscillating feature of the triboelectric tachometer proved in this paper, which will further refine the sensor calibration and shed more light on potential self-powered sensing applications.

## 4. Conclusions

An alternative self-powered triboelectric tachometer with low friction force is presented in this study, which in structure contained a rigid rotor and flexible stator, and in principle utilized the flapping between triboelectric layers on both rigid and flexible parts. The model of this device has been analytically solved, and results show that both the displacement and velocity of the flexible stator tip followed an oscillating style with decreasing peaks as rotation cycle increased, among which tip displacement exhibited a more consistent trend with less fluctuation and deviation. Experimentally, the proposed device was adopted for rotation speed measurement, and the oscillating curve of output voltage versus rotation speed was verified. For the proposed device, the slope of measured rotation speed vs. set rotation speed was 0.9939, demonstrating high linearity. In the future, the proved oscillating relationship could be further refined in terms of contact force duration, and could potentially be applied as calibration criteria for further improving the sensitivity and linearity of triboelectric tachometers.

## Figures and Tables

**Table 1:** Dotted decreasing peaks in the oscillating mean Δ*d*_n_*^NORM^* curve in Figure 4a.

Revolving Cycle *T* (s)	Cycle Ratio *T*/*T_s_*	Mean Δ*d*_n_*^NORM^*
0.033	1.0	9.72
0.066	2.0	4.28
0.100	3.0	2.97
0.133	4.0	2.45
0.165	5.0	2.12
0.199	6.0	1.89
0.231	7.0	1.60
0.267	8.0	1.50
0.300	9.0	1.45
0.333	10.0	1.38
0.366	11.0	1.30

**Table 2:** Distance between highest seven crests in terms of cycle ratio.

Δ*N*_21_	Δ*N*_32_	Δ*N*_43_	Δ*N*_54_	Δ*N*_65_	Δ*N*_76_
0.94	0.60	0.62	0.60	0.53	0.50
